# Association between cerebral perfusion and paediatric postoperative cerebellar mutism syndrome after posterior fossa surgery—a systematic review

**DOI:** 10.1007/s00381-021-05225-5

**Published:** 2021-06-21

**Authors:** Narjes Ahmadian, K. M. van Baarsen, P. A. J. T. Robe, E. W. Hoving

**Affiliations:** 1 Department of Neurology and Neurosurgery, Rudolf Magnus Brain Institute, University Medical Center of Utrecht, 100 Heidelberglaan, G03.126, 3584 CX Utrecht, The Netherlands; 2 Department of Neurology and Neurosurgery, Princess Maxima Center for Pediatric Oncology, Heidelberglaan 25, 3584 CS Utrecht, The Netherlands; 3 Department of Pediatric Neurosurgery, Princess Maxima Center for Pediatric Oncology, Heidelberglaan 25, 3584 CS Utrecht, The Netherlands

**Keywords:** Paediatric postoperative cerebellar mutism syndrome (ppCMS), Posterior fossa syndrome, Cerebral perfusion, Arterial spin labelling (ASL), Dynamic susceptibility contrast (DSC), Single photon emission tomography (SPECT)

## Abstract

**Background:**

Paediatric postoperative cerebellar mutism syndrome (ppCMS) is a common complication following the resection of a cerebellar tumour in children. It is hypothesized that loss of integrity of the cerebellar output tracts results in a cerebello-cerebral “diaschisis” and reduced function of supratentorial areas of the brain.

**Methods:**

We performed a systematic review of the literature according to the PRISMA guidelines, in order to evaluate the evidence for hypoperfusion or hypofunction in the cerebral hemispheres in patients with ppCMS. Articles were selected based on the predefined eligibility criteria and quality assessment.

**Results:**

Five studies were included, consisting of three prospective cohort studies, one retrospective cohort study and one retrospective case control study. Arterial spin labelling (ASL) perfusion MRI, dynamic susceptibility contrast (DSC) perfusion MRI and single photon emission computed tomography (SPECT) were used to measure the cerebral and cerebellar tissue perfusion or metabolic activity. Reduced cerebral perfusion was predominantly demonstrated in the frontal lobe.

**Conclusions:**

This systematic review shows that, after posterior fossa tumour resection, cerebral perfusion is reduced in ppCMS patients compared to patients without ppCMS. Well-powered prospective studies, including preoperative imaging, are needed to ascertain the cause and role of hypoperfusion in the pathophysiology of the syndrome.

## Introduction

Paediatric postoperative cerebellar mutism syndrome (ppCMS) is seen in about 23.5% (range 14.7–47.6%) of children after surgery for a cerebellar tumour [1]. The incidence is found to be even higher in medulloblastomas, 39% [2]. The ppCMS is characterized by mutism or a severe reduction in speech, combined with emotional lability and behavioural changes [3]. It generally occurs within 1 week postoperatively and lasts for weeks to years [4].

The most important risk factors for cerebellar mutism are tumour infiltration into the brainstem or vermis, medulloblastoma pathology, tumour size larger than 4 cm and bilateral injury to the dentate-rubro-thalamic tracts [5–8]. These tracts run from the ipsilateral dentate nucleus through the superior cerebellar peduncle, which cross at the level of the mesencephalon to continue towards the contralateral red nucleus and thalamus [6, 9, 10]. The bilateral damage of this tract is thought to result in a so-called cerebello-cerebral diaschisis, a functional disconnection between the cerebellum and cerebrum. The lack of a functional feedback loop may result in hypofunction of supratentorial areas of the brain [11–15].

Imaging studies, i.e. single-photon emission computerized tomography (SPECT), dynamic susceptibility contrast (DSC) and arterial spin labelling (ASL) MRI studies, claim to have demonstrated the occurrence of cerebral hypoperfusion and cerebral metabolic hypofunction in paediatric patients who developed ppCMS after posterior fossa surgery [16–18]. However, these studies are frequently case reports or small case series. Further, in the majority of studies, the imaging analysis is qualitative rather than quantitative.

To evaluate the evidence for hypoperfusion/hypofunction in the cerebral hemispheres in patients with ppCMS, we systematically reviewed the current literature. We aimed to answer the following PICOT question [19]:

How does surgery for a cerebellar tumour (intervention) influence the cerebral blood flow (outcome) in patients with ppCMS (patients), as opposed to patients without ppCMS (comparison), within 4 weeks postsurgery (time)?

## Methods

The systematic review is conducted according to the PRISMA guidelines [20]. Studies were identified through searches of electronic databases (PubMed, Embase and Cochrane). The search syntax comprised a combination of synonyms and variables for anatomical location, neuropsychological outcome, participant and perfusion/nuclear imaging (as per 16 March 2021; Table 1). Additional studies were identified by scanning reference lists of included articles. Studies were eligible for inclusion if they met the following criteria:

- The study is original.
- The domain is children undergoing posterior fossa tumour surgery.
- Perfusion or nuclear imaging is used.
- Imaging is done within 4 weeks postoperatively
- Postoperative neuropsychological assessments are used for the diagnosis of ppCMS.

### Study selection

Articles were independently screened by two reviewers (NA, KvB), first on title and abstract, and finally on full text to determine eligibility. Disagreements between the two reviewers were resolved by discussion after reading full text.

**Table 1:** Search syntax through PubMed and EMBASE

Anatomical location	Neuropsychological outcome	AND Participants	AND Perfusion/Functional imaging
[ti,ab]	[All fields]	[All fields>	[All fields]
"Cerebel*"[tw] OR "posterior fossa surgery"[tw] OR "posterior cranial fossa surgery"[tw] OR "posterior fossa tumor"[tw] OR "posterior fossa tumour"[tw] OR "posterior cranial fossa tumor"[tw] OR "posterior cranial fossa tumour"[tw] OR "posterior fossa tumors"[tw] OR "posterior fossa tumours"[tw] OR "posterior cranial fossa tumors"[tw] OR "posterior cranial fossa tumours"[tw] OR "Cerebellar Neoplasms/surgery"[mesh] OR "Cranial Fossa, Posterior/surgery"[mesh] OR (("Cranial Fossa, Posterior"[mesh] OR "posterior cranial fossa"[tw] OR "posterior fossa"[tw] OR "Cerebellar Neoplasms"[mesh]) AND ("Surgical Procedures, Operative"[Mesh] OR "surgery"[subheading] OR "surgery"[tw] OR "neurosurgery"[tw] OR "surgical*"[tw] OR "resection"[tw] OR "resect*"[tw] OR "neurosurg*"[tw] OR "operation"[tw] OR "operat*"[tw]	"posterior fossa syndrome"[tw] OR "fossa syndrome"[tw] OR "cerebellar mutism syndrome"[tw] OR "mutism syndrome"[tw] OR "Mutism"[Mesh] OR "mutism"[tw] OR "mutism syndrome"[tw] OR "mute"[tw] OR "mutes"[tw] OR "muted"[tw] OR "muting"[tw] OR "oral-verbal expression"[tw] OR "Speech difficult*"[tw] OR "Speech disorder"[tw] OR "Speech defect"[tw] OR "Speech impair*"[tw] OR "language difficult*"[tw] OR "language impair*"[tw] OR "language disorder"[tw] OR "language defect"[tw] OR "diaschisis"[tw] OR "crossed*"[tw] OR "Speech Disorders"[mesh] OR "Speech Sound Disorder"[Mesh] OR "speech"[tw] OR "speech*"[tw] OR "language"[tw] OR "speaking"[tw] OR "speak*"[tw	"Child*"[tw] OR "child"[mesh] OR "infant*"[tw] OR "pediatric*"[tw] OR "paediatric*"[tw] OR "pediatrics"[mesh] OR "girl"[tw] OR "girls"[tw] OR "girlhood"[tw] OR "boy"[tw] OR "boy"[tw] OR "boyhood"[tw]	"Arterial spin labeling"[tw] OR "ASL"[tw] OR "Spin Labels"[Mesh] OR "perfusion weighted mri"[tw] OR "PWI"[tw] OR "perfusion weighted imag*"[tw] OR "Perfusion Imaging"[Mesh] OR "Perfusion Imaging"[tw] OR "dynamic susceptibility contrast"[tw] OR "DSC"[tw] OR "mr perfusion"[tw] OR "Magnetic Resonance Perfusion"[tw] OR "Magnetic Resonance Angiography"[Mesh] OR "dynamic contrast enhanced"[tw] OR "DCE"[tw] OR "hypoperfusion"[tw] OR "decreased perfusion"[tw] OR "altered perfusion"[tw] OR "frontal lobe perfusion"[tw] OR "temporal lobe perfusion"[tw] OR "parietal lobe perfusion"[tw] OR "occipital lobe perfusion"[tw] OR "cerebral perfusion"[tw] OR "frontal lobe hypoperfusion"[tw] OR "temporal lobe hypoperfusion"[tw] OR "parietal lobe hypoperfusion"[tw] OR "occipital lobe hypoperfusion"[tw] OR "Tomography, Emission-Computed, Single-Photon"[Mesh] OR "SPECT"[tw] OR "Single Photon Emission Tomography"[tw] OR "functional mri"[tw] OR "fmri"[tw] OR "functional magnetic"[tw] OR "Magnetic Resonance Imaging"[Mesh] OR "Blood flow"[tw] OR "blood oxyg*"[tw] OR "hemodynamic response"[tw] OR "bold contrast"[tw] OR "Blood-oxygen level dependent contrast"[tw] OR"Neuroimaging"[Mesh] OR "neuroimag*"[tw] OR "brain ischemia"[mesh]

### Risk of bias evaluation

The risk of bias in the individual studies was assessed by two reviewers (NA, KvB) using the Quality in Prognosis Studies tool (QUIPS) developed by Cochrane [35]. The QUIPS tool was based on the following key domains: study participation, prognostic factor measurement, outcome measurement, study confounding, statistical analysis and reporting. The overall risk of bias was judged as low, moderate or high based on the following criteria: (1) low if there was a low risk of bias in all key domains, or only one unclear risk of bias in one key domain, (2) unclear/moderate risk of bias if there was an unclear risk of bias for two key domains and (3) high risk of bias if there was a high risk of bias for one or more key domains or unclear risk of bias for more than two key domains.

### Data extraction

Data were extracted from the studies on the number of included patients (with and without ppCMS), patient age and gender, tumour location, type of pathology, type of perfusion/nuclear imaging, time interval between surgery and postoperative scan, postoperative neuropsychological tests and outcome measures (e.g. mean deviation (MD) with 95% confidence interval (CI)). If the data were not reported, outcome measures were calculated based on other reported data or based on the qualitative assessment of the nuclear specialist. The standard unit for measurement of cerebral blood flow (CBF) is ml blood /min/100 g tissue [21]. As there are no cut-off values for normal cerebral perfusion in children, the definition of hypoperfusion was at the discretion of the authors.

Any data on possible confounding factors, such as the amount of blood loss during the surgery, total volume of fluid infusions and mean body temperature, were also collected [8].

## Results

**Fig. 1:**
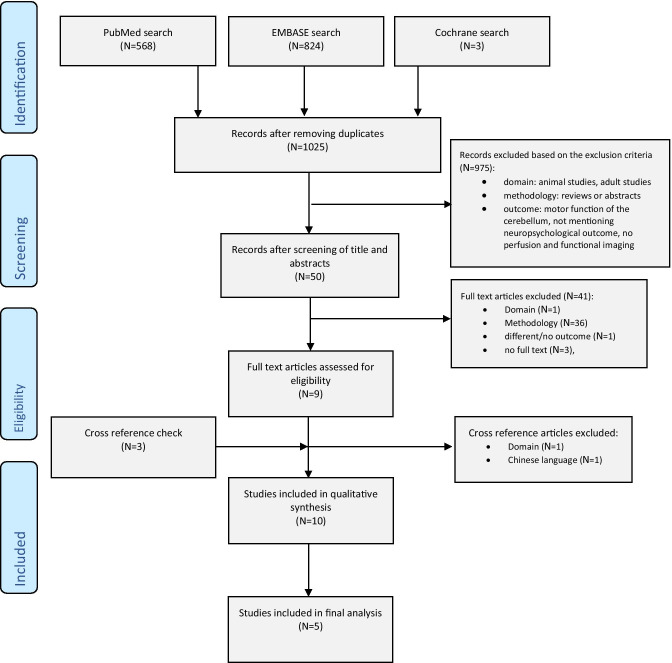
PRISMA Flow Diagram

The search identified 1125 articles from PubMed and Embase databases, of which 50 fulfilled the inclusion criteria, based on title and abstract. One study was found through cross reference checking (Fig. 1). After screening full text, five studies were included in this review, consisting of three prospective cohort studies, one retrospective cohort study and one retrospective case control study (Table 2).

**Fig. 2:**
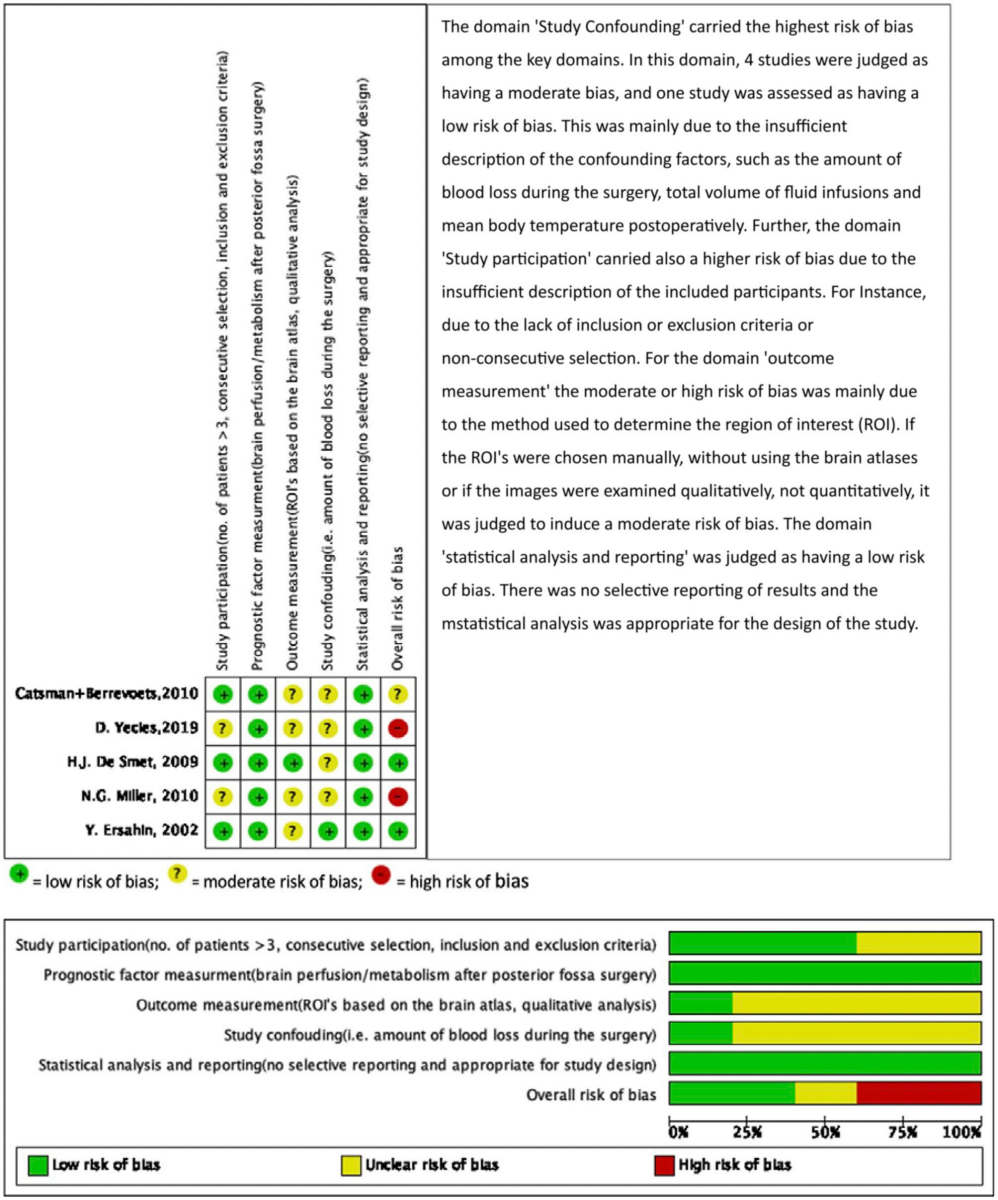
Risk of bias according to the QUIPS tool

The baseline characteristics of the patients included in these five studies are shown in Table 2. The total number of patients was 80. Age was reported with a median of 7 years (range 2–18 years). In the majority of the studies, more boys were included than girls. One study used ASL perfusion MRI, one study used DSC perfusion MRI and three other studies used SPECT to measure the cerebral and cerebellar tissue perfusion/metabolic activity. The time to postoperative imaging follow-up was within 4 weeks. Pre-operative perfusion/nuclear imaging was only acquired in one out of a total of 80 patients [22]. Medulloblastoma was the most frequent tumour pathology among the patients, in the ppCMS as well as the non-ppCMS group, and tumours were mainly located in the fourth ventricle and the cerebellar vermis.

**Table 2:** Baseline characteristics of patients in the selected articles

Authors	Study design	PreopImaging*	PostopImaging*	Time to Postop imaging(weeks)	ppCMS +					ppCMS -				
					N	Age(mean)	Gender(M/F)	Tumour location	Pathology	N.	Age(mean)	Gender(M/F)	Tumour location	Pathology
Ersahin et al. [24]	PS C	-	SPECT	1	3	3.3	1M/2F	4V, V	2MB,1EP	11	6.6	5M/6F	V, 4V, CH	5PA, 3EP, 1MB, 1DT, 1CPP
De Smet et al. [22]	PS C	-	SPECT	1-4	4	7.5	4M	V	2MB, 1PA, 1EP	1	14	1M	V	1PA
Catsman-Berrevoets et al. [25]	PS C	-	SPECT	1-4	13	9.3	9M/4F	V	3PA, 10MB	2	8.5	2M	V	2MB
Yecies et al. [23	RS C	-	ASL	1	14	7.1	8M/6F	4V, V	14MB	10	NM	NM	4V, V	10MB
Miller et al. [26]	RS CC	-	DSC	3-4	11	7.1	NM	NM	MB, ATRT or PN	11	7.33	NM	NM	NM

*N* number of subjects, *NM* not mentioned, *NA* not applicable, *M* male, *F* female *RS* retrospective, *PS* prospective, *CR* case report, *CC* case control, *C* cohort. *ASL* Arterial Spin Labelling, *DSC* Dynamic Susceptibility Contrast, *SPECT* Single Photon Emission Computed Tomography

*PA* pilocytic astrocytoma, *MB* medulloblastoma, *EP* anaplastic ependymoma, *ATRT* atypical teratoid/rhabdoid, *PN* primitive neuroectodermal, *DT* dermoid tumour, *SB* spongioblastoma, *CPP* choroid plexus papilloma

*V* vermis, *4V* fourth ventricle, *LC* left cerebellar, *CH* cerebellar hemisphere

* nuclear or perfusion imaging

Based on the evaluation of 6 key domains, 3 studies were judged to have a low risk of bias and 2 studies had a high risk of bias. This was largely due to the fact that there was a lack of information on patient selection, no correction for confounding factors and heterogeneity in outcome measurements (Fig. 2). Cerebral perfusion was evaluated using different imaging and software techniques with a large variability in outcome measures. For instance, in the ASL study of Yecies et al., the Regions of Interest (ROIs) were acquired manually without using atlases [23]. Further, in some studies, the CBF measurements were acquired qualitatively, not quantitatively; hence, the measurements might have been subject to the investigator’s interpretation [24].

Most of the studies assessed brain tissue perfusion in the following anatomical areas: frontal lobe, parietal lobe, temporal lobe, occipital lobe, thalamus, cerebellum and proximal efferent cerebellar pathways (Table 3). Brain perfusion in the corpus cinguli and brainstem was evaluated in only one study.

In all included studies, hypoperfusion was demonstrated almost exclusively in children with ppCMS when compared to controls. Reduced cerebral perfusion was predominantly demonstrated not only in the frontal lobe, but also in the temporal lobe, parietal lobe, occipital lobe, thalamus and the cerebellum. Hypoperfusion was reported to occur either bilaterally or in the right hemisphere, except in one study that showed hypoperfusion in the left cingulate gyrus in the language-dominant left or bilateral frontal lobe and in the left cingulate gyrus during the mutism phase [22].

The quantitative analysis of Yecies et al. demonstrated a significant difference in right frontal lobe perfusion in ppCMS patients compared to the controls (mean deviation −12.70 [CI −23.75 to −1.65], p = 0.046) (Table 3) [23]. There was also hypoperfusion in the left frontal lobe (mean deviation −12.50 [CI −25.34 to 0.34], p = 0.092) in ppCMS patients as compared to the controls, but this difference was not statistically significant.

Increased perfusion, or hyper-perfusion, was not reported by any of the studies.

**Table 3:** Perfusion of different brain regions

ppCMS +										ppCMS -							
Author	Imaging	CBF	FRO	PAR	TEM	OCC	CER	THA	Other	CBF	FRO	PAR	TEM	OCC	CER	THA	Other
Ersahin et al., 2002^24^	SPECT	NA	B-	B-	n	n	R-	n	NA	NA	n	n	n	n	n	n	NA
De Smet et al., 2009^22^	SPECT	NA	B-	B-	B-	R-	B-	B-	L- CC	NA	R-	n	n	L-	L-	n	n
Catsman-Berrevoets et al., 2010^25^	SPECT	NA	B-	n	B-	B-	B-	n	BS-	NA	n	n	n	n	n	n	n
Yecies et al., 2019^23^	ASL	R:37.00 ±13.46, L: 41.00 ± 17.36	B-	n	n	n	n	n	NA	R:49.70 ± 13.72, L: 53.50 ± 14.62	n	n	n	n	n	n	NA
Miller et al., 2010^26^	DSC	47.3 to 54.5	B-	R-	R-	n	n	n	NA	55.9 to 67.6	n	n	n	n	n	n	NA

*NA* not applicable, *NM* not mentioned, *ASL* Arterial Spin Labelling, *DSC* Dynamic Susceptibility Contrast, *SPECT* Single Photon Emission Computed Tomography, *R* right, *L* left, *B* bilateral, *CBF* mean cerebral blood flow in ml/min/100g, *CER* cerebellum, *THA* thalamus, *FRO* frontal, *PAR* parietal, *TEM* temporal, *OCC* occipital , *CC* corpus cinguli, *BS* brainstem, *n* normal

## Discussion

This systematic review shows that, after posterior fossa tumour resection, cerebral perfusion is reduced in ppCMS patients compared to patients without ppCMS. All included studies show predominantly bilateral frontal lobe hypoperfusion in children with ppCMS.

ASL perfusion imaging identified decreased frontal lobe blood flow in the immediate postoperative period in ppCMS group as compared to non-ppCMS patients. In addition, this hypoperfusion resolved after the period of mutism [23].

The DSC study showed a decrease in cerebral blood flow within frontal, right parietal and temporal areas. Further, a global cerebral cortical hypoperfusion is seen in the ppCMS group as compared to controls [26]. Only one study found hypoperfusion in the non-ppCMS group, but in different regions and only in one patient [22].

Although case reports were excluded from our systematic analysis, they do support our findings. All case reports demonstrated hypoperfusion of the frontal lobes bilaterally [16, 18, 27]. Further, Sagiuch et al. and Watanabe et al. reported additional hypoperfusion bilaterally in the cerebellum and the thalami. Sagiuch et al. and Germano et al. also reported a reduced cerebral perfusion in the language dominant left temporal lobe. The clinical improvement after the period of mutism coincided with improvement in SPECT and ASL images [16, 18].

### Imaging modalities

Arterial spin labelling (ASL) has several advantages when compared to DSC and SPECT imaging. ASL is a magnetic resonance imaging technique that enables the measurements of cerebral blood flow non-invasively by magnetically labelling inflowing blood at the tissue level [28, 29]. It does not require the administration of an intravenous contrast agent and is thus easily repeatable. It does require a high signal to noise ratio, which is better in children compared to adults because of their higher cerebral blood flow [31, 32].

Contrary to ASL, both DSC and SPECT imaging are invasive imaging techniques for both adult and paediatric patients. SPECT requires intravenous administration of technetium-99 m-labelled d, l-hexamethyl propylene amine oxime (Tc99M-HM-PAO), and DSC imaging requires bolus intravenous injection of a gadolinium-based contrast agent for the measurement of its regional cerebral uptake [11, 16]. Therefore, ASL is the technique of choice for (repeated) perfusion measurement in young children [29, 30].

### Limitations

None of the included studies in this review conducted pre-operative perfusion or nuclear imaging, in order to compare with the postoperative findings. Preoperative comparison is important for a correct interpretation of the postoperative findings. For instance, hematocrit effects in patients with anaemia can confound the interpretation of CBF changes measured using ASL MRI [33]. More importantly, a perfusion deficit that was already present preoperatively cannot be ascribed to the surgery and cannot explain the pathophysiology of the postoperative cerebellar mutism syndrome.

Pre-operative perfusion imaging was conducted in only one case report [18]. In this single case, postoperative ASL perfusion MRI revealed hypoperfusion bilaterally in the frontal lobes, the thalamus and the cerebellar hemispheres when compared to preoperative ASL [18].

Further, none of the studies mentioned any confounding factors. A higher mean body temperature on the first postoperative days might increase metabolic stress, while the area of surgery and the brain tissue surrounding the site of surgery is in an already critical metabolic status [8]. Further, fluid balance, including volume of blood loss and total volume of liquid infusions, might affect the CBF [8].

Another limitation was the large heterogeneity among the included studies that may have influenced the results of this systematic review.

### Level of evidence

According to the Oxford Centre for Evidence Based Medicine [34], this systematic review offers a “Level 4” evidence despite the systematic approach and the strict selection of articles.

### Clinical importance

Even though there seems to be convincing evidence for bilateral frontal perfusion deficits in ppCMS patients as compared to non-ppCMS patients, without a preoperative comparison, this does not fully support the hypothesis of cerebello-cerebral diaschisis as pathophysiological explanation of the syndrome. Recognizing the ppCMS and understanding its pathophysiology is important for education of patients and families, to develop treatment strategies and rehabilitation programs and, eventually, to prevent its occurrence.

Future research should aim for a large prospective observational cohort study including non-invasive imaging techniques such as ASL both pre- and postoperatively, allowing quantitative measurement of the CBF in children with and without ppCMS. This will give valuable information to further unravel the pathophysiology of this devastating syndrome.

## Conclusion

This systematic analysis of the literature shows that bilateral frontal lobe hypoperfusion is predominantly seen in patients with ppCMS as compared to those without. Well-powered prospective studies, including preoperative imaging, are needed to ascertain the cause and role of hypoperfusion in the pathophysiology of the syndrome.
